# Changes in Antibody Seroprevalence of Seven High-Risk HPV Types between Nationwide Surveillance Studies from 1995–96 and 2006–07 in The Netherlands

**DOI:** 10.1371/journal.pone.0048807

**Published:** 2012-11-12

**Authors:** Mirte Scherpenisse, Madelief Mollers, Rutger M. Schepp, Hein J. Boot, Chris J. L. M. Meijer, Guy A. M. Berbers, Fiona R. M. van der Klis, Hester E. de Melker

**Affiliations:** 1 Laboratory for Infectious Diseases and Screening, National Institute of Public Health and the Environment, Bilthoven, The Netherlands; 2 Department of Epidemiology and Surveillance, National Institute of Public Health and the Environment, Bilthoven, The Netherlands; 3 Department of Pathology, VU University Medical Centre, Amsterdam, The Netherlands; The Catalan Institute of Oncology (ICO), Spain

## Abstract

**Objective:**

This study evaluates trends in antibody seroprevalences of seven high-risk human papillomavirus (hr-HPV) serotypes (HPV16, 18, 31, 33, 45, 52, and 58) between the 1995–96 and 2006–07 sero-surveys among the Dutch general population in the pre-vaccination era.

**Methods:**

Serum samples of men and women (0–79 years of age) from two cross-sectional population-based serosurveillance studies performed in 1995–96 (n = 3303) and 2006–07 (n = 6384) were tested for HPV-specific antibodies in a VLP-based multiplex immunoassay.

**Results:**

HPV16-specific antibody seroprevalence increased during adolescence and shifted to younger ages in the 2006–07 survey compared to the 1995–96 survey. This step-up in HPV16 seroprevalence was most pronounced in women, while a more gradual increase was observed in men. Also in cohorts older than 49 years, HPV16 seroprevalence was higher in 2006–07 as compared to 1995–96 survey. A higher overall seroprevalence in individuals older than 15 years of age was found for HPV16, 18, 31 and 45 in 2006–07 as compared to 1995–96. For HPV33, 52 and 58 seroprevalences were comparable over this 11-year time period. Seropositivity for one or more HPV types was significantly higher in 2006–07 (23.1%) than in 1995–96 (20.0%) (p = 0.013). Multi-seropositivity increased from 7.1% in 1995–96 up to 10.2% in 2006–07 (p<0.0001). Differences in HPV seropositivity for at least one of the seven HPV types between both surveys could be explained in addition to demographic characteristics (age, sex, urbanization degree and ethnicity), also by changes in sexual behaviour (marital status, age of sexual debut and ever reported an STI).

**Conclusion:**

The observed increase in particular HPV16 seroprevalence could be due to changes in sexual behaviour over the years, and especially in age of sexual debut. Seroprevalence studies provide insight into the distribution of HPV types and infection dynamics in the general population over time, which is important to assess the impact of HPV-vaccination.

## Introduction

Human papillomavirus (HPV) consists of a large family of more than 120 HPV genotypes of which 40 types are oncogenic [1]. These oncogenic HPV types can cause cervical cancer, other genital related cancers and oro-pharyngeal cancers. HPV infections are the major cause of cervical cancer and in 99.7% of the cases HPV DNA can be identified [2]. The two most important oncogenic HPV genotypes detected in cervical cancer are HPV16 and 18 [3].

HPV is a sexually transmitted virus and the highest HPV antibody seroprevalence is found among individuals 20–40 years of age with a decreasing seroprevalence in elderly [4], [5]. Age-related trends in seroprevalence might be due to HPV incidence, cohort effects and waning of detectable antibody levels [4]. Women were found HPV seropositive more often than men [4], [6], [7]. Infections in men often involve keratinized epithelium that may be less likely to induce a humoral immune response than infection of mucosal epithelium [7].

Because HPV-specific antibodies are not often observed in transient infections, seroconversion is more strongly associated with persistent HPV infections [8], [9]. Measurable HPV-specific antibody responses in serum develop in approximately 50–70% of individuals infected with HPV, probably due to the fact that HPV is able to evade the host immune system [10], [11]. Serological HPV responses are a measure of past HPV exposure as in naturally infected individuals HPV antibody concentrations persist for many years [12], [13].

Currently, comparisons between studies on trends in serological hr-HPV prevalence over time are limited because most studies are focused on DNA prevalence or incidence of cervical intraepithelial neoplasia (CIN) in women [14], [15], [16], [17]. We have examined changes in antibody seroprevalence between 1995–96 and 2006–07 surveys in men and women in The Netherlands for HPV serotypes 16, 18, 31, 33, 45, 52, and 58. These data will provide more information about the number of HPV exposures over time and possible changes in HPV serotypes within this time period. In addition, these data serves as a baseline before the implementation of the HPV vaccine in the Dutch national immunization program in 2010 and are thus valuable in assessing the impact of the HPV vaccination program on a population level.

## Methods

### Ethics statement

The study proposal was approved by the Medical Ethics Testing Committee of the foundation of therapeutic evaluation of medicines (METC-STEG) in Almere, The Netherlands (clinical trial number: ISRCTN 20164309). A written informed consent was obtained from all participants and for those below 18 years of age also from the parents, care takers or guardians.

### Study populations

In The Netherlands, serum samples from two cross-sectional population-based serosurveillance studies performed from October 1995 to December 1996 and from February 2006 to June 2007 were available. Participants, women, men and children, of both studies were 0–79 years of age. The participation rates for the 1995–96 and 2006–07 surveys were 55% and 32%, respectively. Study designs have been described earlier [18], [19]. Briefly, the participants were asked to fill in a questionnaire and to provide a blood sample. Both questionnaires included data for instance on demographic characteristics, ethnicity (first and second generation migrants), vaccination history and sexual behaviour. The questionnaire used in 1995–96 was extended in the 2006–07 survey with more questions about sexual behaviour and migrants. Information related to sexual behaviour was only available from participants older than 15 years of age in the 1995–96 study and from participants older than 14 years of age in the 2006–07 study.

### Serological measurements

Serum samples of both surveys were stored at −80°C until analysis. For the measurement of HPV-specific IgG serum antibodies against L1 virus-like-particles (VLP) of HPV16, 18, 31, 33, 45, 52, and 58, a VLP-based multiplex immunoassay (MIA) was used as previously described [20]. GSK (GlaxoSmithKline Biologicals S.A., Rixensart, Belgium) kindly supplied the HPV-VLPs. Briefly, VLPs were conjugated to seven distinct fluorescent microspheres via amine coupling. Serum samples were 1/50 diluted and incubated with the VLP-coupled microspheres. HPV-specific IgG serum antibodies were detected using a secondary goat anti-human phycoerythrin-labelled antibody. Four in-house control sera and an in-house standard were used on each plate. The in-house standard (IVIG, lot LE12H227AF, Baxter) was calibrated against reference serum of GSK for all the seven HPV types. HPV-specific IgG antibodies were analyzed using the Bioplex system 200 with Bioplex software (Bio-Rad Laboratories, Hercules, CA). Samples were assumed to be seropositive above cut-offs determined previously with this assay: 9, 13, 27, 11, 19, 14, and 31 Luminex Units/ml (LU/ml) for HPV16, 18, 31, 33, 45, 52, and 58, respectively.

### Statistical analysis

Data analyses were conducted using SAS version 9.2 and Graphpad version 5.0. Age-specific seroprevalences were calculated and weighted. Weights (sex, age, ethnic origin and urbanization degree) for the 1995–96 survey were determined proportional to the Dutch reference population on January 1^st^ 1996 and for the 2006–07 survey on January 1^st^ 2007.

To test whether any trends could be explained by differences in risk factors between the two serosurveys we performed a logistic regression analysis on a pooled dataset of both surveys for individuals older than 15 years of age. Only persons with information about all risk variables were included in the analyses (74%). We included the following variables: age, sex, urbanization degree, ethnicity, marital status and sexual behaviour. Besides odds ratios, we calculated unadjusted and adjusted probabilities and converted these to percentages [21] to show the actual reduction in seroprevalence between both surveys. We compared unadjusted HPV seroprevalences (study parameter) with seroprevalences adjusted for demographic characteristics (age, sex, urbanization degree and ethnicity) and then demographic characteristics with sexual risk behaviour (marital status, age of sexual debut and a self-reported history of sexual transmittable infections (STI). The risk factor analysis was carried out for combined HPV seropositivity, in which the risk factor analysis included data on seropositivity for at lease one of the seven HPV types. In addition, the risk factor analysis was assessed for seropositivity of each HPV type separately.

Statistically significant differences (p<0.05) for HPV combinations or HPV seroprevalences were analyzed using a Chi-square test and differences between antibody concentrations of seropositive individuals were analyzed using a Mann-Whitney test.

## Results

### Characteristics of the serosurveillance studies

We tested 3303 serum samples from the 1995–96 survey (48% men and 52% women and 6386 samples from the 2006–07 survey (46% men and 54% women). In the 1995–96 survey the cohorts older than 15 years of age contained more individuals with the Dutch nationality, were married and less self-reported history of STI (Table 1). The mean age of sexual debut in persons younger than 26 years was higher in the 1995–96 survey (17.5 years, SD 17.1–17.9) as compared to the 2006–07 survey (16.9 years, SD 16.6–17.2). In both surveys women were younger at first sexual intercourse (1995–96: 17.2 years, SD: 16.7–17.6, 2006–07: 16.6 years, SD: 16.3–16.8) than men (1995–96: 17.9 years, SD: 17.5–18.5, 2006–07: 17.3 years, SD: 16.7–17.8).

**Table 1:** Study characteristics of the 1995–96 and 2006–07 survey of individuals older than 15 years of age.

	1995–96 survey	2006–07 survey	
Variable	N (%)	N (%)	P-value
**Age**			
16–19	103 (5)	160 (4)	0.0425
20–24	188 (9)	364 (8)	
25–29	193 (9)	348 (8)	
30–39	364 (17)	715 (17)	
40–49	330 (15)	641 (15)	
50–59	382 (17)	714 (16)	
60–69	333 (15)	799 (18)	
>70	295 (13)	593 (14)	
**Sex**			
Male	996 (46)	1864 (43)	0.0535
Female	1192 (54)	2470 (57)	
**Ethnicity**			
Dutch	1988 (91)	3544 (82)	<0.0001
First generation migrants	94 (4)	498 (11)	
Second gen. migrants	106 (5)	292 (7)	
**Marital status**			
Married	1372 (63)	2547 (59)	<0.0001
Living together	180 (8)	469 (11)	
Not married	406 (19)	851 (20)	
Divorced	88 (4)	240 (6)	
Widow	142 (6)	227 (5)	
**Ever had sexual intercourse**			
No	107 (5)	177 (4)	0.0531
Yes	1898 (95)	4001 (96)	
**Number of sex partners**	last 12 months	last 6 months	
0	370 (19)	662 (19)	N/A
1–2	1506 (79)	2736 (80)	
>2	26 (1)	20 (1)	
**Age at first sexual intercourse**			
<17	236 (15)	677 (21)	<0.0001
17–19	575 (36)	1251 (39)	
>19	789 (49)	1307 (40)	
Mean (SD), years*	17.5 (17.1–17.9)	16.9 (16.6–17.2)	
**Ever had STI**			
no	1821 (98)	3612 (94)	<0.0001
yes	39 (2)	211 (6)	

**Note**: Persons are ≥16 years of age, 1995–96 survey n = 2188, 2006–07 survey n = 4334,

* Persons <26 years, N/A: not applicable.

### Comparison of HPV antibody seroprevalences in 1995–96 and 2006–07

**Figure 1:**
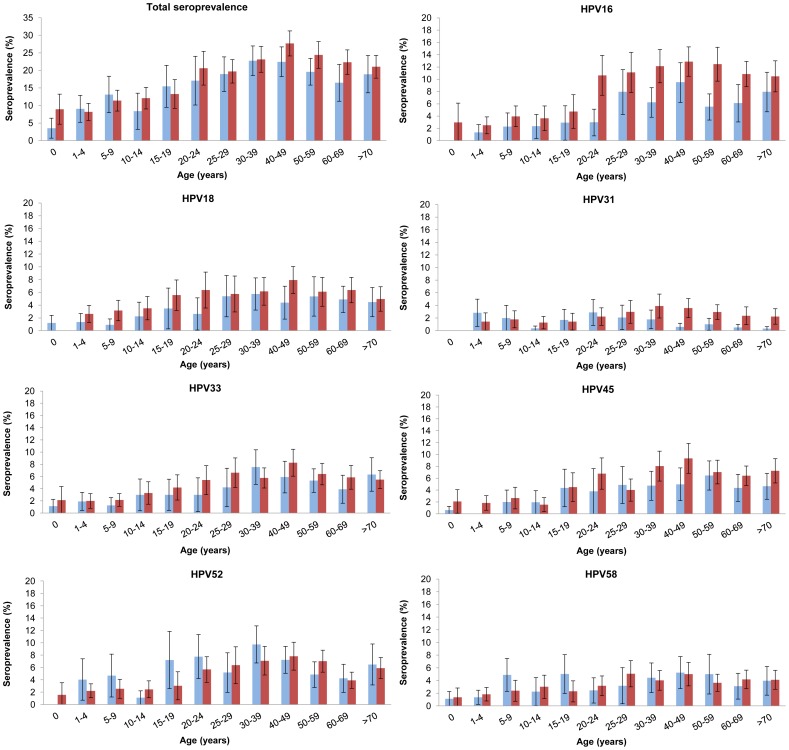
Trends in antibody seroprevalence. Total seroprevalence for any HPV serotype and overall seroprevalence for the seven hr-HPV serotypes tested between the 1995–96 (blue bars) and 2006–07 (red bars) surveys.

**Figure 2:**
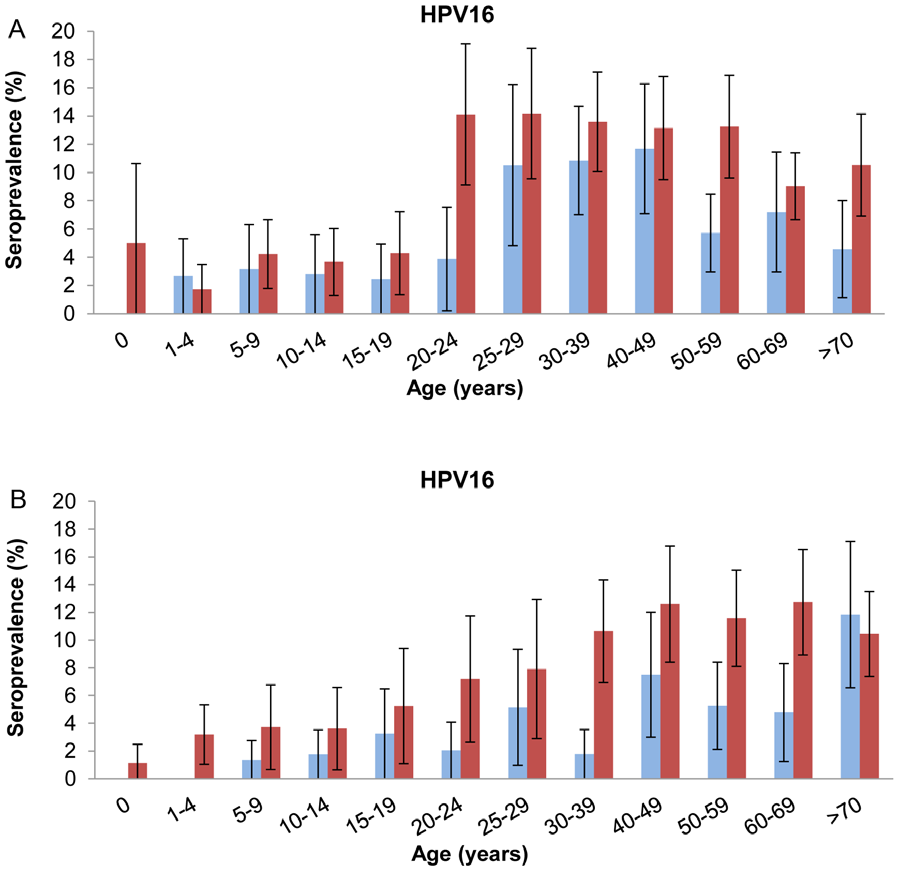
Comparison of trends in HPV16 antibody seroprevalence. Seroprevalence among women (A) and men (B) in the 1995–96 (blue bars) and 2006–07 (red bars) surveys.

The total age-specific antibody seroprevalence for any HPV type started to increase in the cohort of 15–19 years up to 30–39 years in 1995–96 and to 40–49 years in 2006–07 (Figure 1). In the older age groups (>49 years) a significant HPV-specific decreasing trend was observed in 2006–07 (p = 0.013). Nevertheless, a higher total HPV seroprevalence was observed in 2006–07 compared with 1995–96 for individuals older than 40 years of age. In addition, a gradual rise in total HPV seroprevalence was observed from birth up to the age cohorts of 15–19 years. HPV16 seroprevalence was higher in 2006–07 as compared to 1995–96 in all age groups. Interestingly, a clear step-up in HPV16 seroprevalence was observed among the adolescent/young adult cohorts, which occurred at an earlier age in 2006–07 (age cohort 15–19 years) compared with 1995–96 (age cohort 20–24 years) (Figure 2A). This step-up in HPV16 seroprevalence was most pronounced in women, while a more gradual increase with age in HPV16 seroprevalence was found in men in both surveys (Figure 2B). For the other 6 HPV types also a more gradual increase in age-specific seroprevalence was observed with highest seroprevalences in individuals aged 30–59 years (Figure 1). For HPV33 and 45 higher age-specific seroprevalences were found in 2006–07 compared with 1995–96 while HPV52 was slightly higher in the 1995–96 survey.

Focusing on individuals older than 15 years of age, the most prevalent HPV-specific antibodies in 2006–07 were directed against HPV16 (11.5%) followed by HPV45 (7.2%), while in 1995–96 antibodies against HPV52 (6.9%) and HPV16 (6.6%) were most prevalent (Table 2). In 2006–07, significantly higher overall seroprevalences were found for HPV serotypes 16 (p<0.0001), 18 (p = 0.015), 31 (p = <0.0001) and 45 (p = 0.006) compared with 1995–96, while for the other three HPV serotypes (33, 52 and 58) seroprevalences were comparable. A combination of HPV16 and/or HPV18 seropositivity was also higher in 2006–07 (13.2%) than in 1995–96 (9.6%) (p<0.0001) (Table 3). Moreover, the percentage of individuals who were seropositive for one or more HPV types was higher in 2006–07 (23.1%) than in 1995–96 (20%) (p = 0.013). Also seropositivity for ≥2 or ≥3 HPV types significantly increased from 7.1% or 3.4% in 1995–96 to 10.2% or 5.1% in 2006–07 (p<0.0001, p = 0.002), respectively. Only a small percentage of individuals (1.9% in 1995–96 and 3.0% in 2006–07 survey) was seropositive for ≥4 HPV serotypes in both surveys gradually decreasing to 0.2% in the 1995–96 survey and 0.4% in the 2006–07 survey for 7 HPV types.

**Table 2:** Overall antibody seroprevalences of seven hr-HPV serotypes of individuals older than 15 years of age in the 1995 and 2006–07 survey.

	1995–96		2006–07		
	%	(95%CI)	%	(95%CI)	p-value
**Clade α9**					
**HPV16**	6.6	(5.5–7.6)	11.5	(10.4–12.5)	<0.0001
**HPV31**	1.3	(0.7–1.8)	3.0	(2.3–3.6)	<0.0001
**HPV33**	5.5	(4.4–6.5)	6.3	(5.5–7.1)	0.205
**HPV52**	6.9	(5.8–8.0)	6.3	(5.6–6.9)	0.330
**HPV58**	4.3	(3.2–5.4)	4.1	(3.5–4.8)	0.768
**Clade α7**					
**HPV18**	4.8	(3.7–5.8)	6.4	(5.6–7.2)	0.015
**HPV45**	5.0	(3.8–6.2)	7.2	(6.2–8.2)	0.006

**Note**: 1995–96 survey n = 2188, 2006–07 survey n = 4334.

**Table 3:** Combinations of overall HPV antibody seropositivity of individuals older than 15 years of age in the 1995–96 and 2006–07 survey.

	1995–96		2006–07		
	%	(95%CI)	%	(95%CI)	p-value
**HPV16 and 18**	2.3	(1.0–3.6)	4.7	(4.1–5.3)	0.186
**HPV16 or 18**	8.0	(6.7–9.2)	8.5	(7.5–9.4)	0.517
**HPV16 and/or 18**	9.6	(8.4–10.9)	13.2	(12.1–14.3)	<0.0001
**Positive ≥1 HPV types**	20.0	(17.9–22.1)	23.1	(22.1–24.5)	0.013
**Positive ≥2 HPV types**	7.1	(6.1–8.1)	10.2	(9.3–11.1)	<0.0001
**Positive ≥3 HPV types**	3.4	(2.6–4.2)	5.1	(4.3–6.0)	0.002

**Note:** 1995–96 survey n = 2188, 2006–07 survey n = 4334.

### HPV type-specific antibody concentrations among seropositive individuals

Although a higher HPV16 seroprevalence was found in 2006–07 compared with 1995–96, the age-specific HPV16 geometric mean concentrations (GMCs) of seropositive individuals was comparable in all age cohorts between both studies (Figure S1). For HPV18, higher GMCs were found in 2006–07 as compared to 1995–96 reaching significance in the age cohorts 1–4 years (p = 0.02), 50–59 years (p = 0.04), and 60–69 years (p = 0.02). For HPV types 33, 45 and 52 significant higher GMCs were found in 2006–07 as compared to 1995–96 for the age cohorts 1–4 years (HPV33, p = 0.01), 40–49 years (HPV45, p = 0.0004), 50–59 years (HPV33, p = 0.04), 60–69 years (HPV52, p = 0.001) and 70–79 years (HPV45, p = 0.04). Comparable GMCs of seropositive individuals were found for HPV31 and 58 between 1995–96 and 2006–07 in all age-cohorts.

### Pooled risk factor analysis associated with HPV antibody seropositivity in the 1995–96 and 2006–07 surveys

HPV seropositivity for at least one of the seven HPV types in individuals older than 15 years of age before adjustment of any variables was 20.7% in the 1995–96 study and 23.5% in the 2006–07 study (OR:1.2, 95%CI 1.0–1.4). After pooling both surveys, adjustment of demographic characteristics (age, sex, urbanization degree and ethnicity) resulted in a smaller difference in HPV seroprevalence between the 1995–96 survey (21.0%) and 2006–07 (23.2%) survey (OR: 1.1, 95%CI 1.0–1.3). The difference between both surveys decreased even further with adjustments for demographic characteristics and sexual risk factors (marital status, age of sexual debut and a self-reported history of STI). The HPV seroprevalence of the 1995–96 survey (22.2%) and the 2006–07 survey (22.8%) only differed by 0.6% (OR: 1.0, 95%CI 0.9–1.2).

HPV types 16, 18, 31, and 45 showed a significant difference between the 1995–96 and 2006–07 survey before adjustment of any variables. The risk factor analysis for seropositivity of each HPV type separately was comparable with the risk factor analysis of combined HPV seropositivity (seropositive for at least one of the seven HPV types), in which a decrease in ORs after adjusting for demographic characteristics and sexual risk factors was observed. However, even after adjustment of demographic characteristics and sexual risk factors the difference for HPV16 (OR: 1.5, 95%CI 1.2–2.0) and HPV31 (OR: 1.7, 95%CI 1.1–2.7) between the two surveys remained statistically significant.

## Discussion

Several observations from this study indicate that the situation on HPV incidence has changed in The Netherlands. First, the overall antibody seroprevalence of seven hr-HPV types has increased, in the age cohorts older than 15 years of age, 3.1% in 2006–07 as compared to the serosurveillance study 11 years earlier due to a significant higher seroprevalence for HPV16, 18, 31 and 45. Secondly, the clear-cut rise in HPV16 seroprevalence in particular women has shifted to younger aged cohorts (from 25–29 to 20–24 years). Finally, seropositivity for HPV16/18 combinations, for one or more types, and for multiple HPV serotypes has also significantly increased during the 11-year time interval.

Comparable HPV-specific antibody levels of seropositive individuals were observed between the 1995–96 and 2006–07 survey, suggesting that factors involved in the generation of HPV-specific antibodies have not changed over time. The increase in HPV seroprevalence, particularly for HPV16, between the two time periods could be due to an increase in HPV infection and/or HPV circulation [22]. However, Kibur et al. did not found major differences in the age-specific HPV16 seroprevalence and incidence among Finnish primiparous women between the 1980s and 1990s [23]. Interestingly, in the late 1990s HPV16 antibody seroprevalence increased in Finland especially in women up to 31 years of age [24]. In line with this, an increase in cervical cancer cases among women below 55 years of age was observed [25]. Also higher frequencies of multiple HPV infections, high-risk HPV infections and changes in the distribution of HPV types was observed in women diagnosed with CIN over the period 1985–2007, with major changes over the last ten years [15]. A Danish study showed that HPV-associated anal cancers increased between 1998–2008, especially in women, while non-HPV associated cancers remained relatively constant [26]. In The Netherlands, an increase in CIN3 lesions in the cervical screening registry has been observed since 2005. This increase in CIN3 might be due to more HPV circulation, however, an effect of changing screening algorithms can not be ruled out [27], [28].

Over the years, higher population mobility in recent years, including immigration of populations who are at higher risk for HPV seropositivity and also changes in sexual behaviour, for instance a younger age of sexual debut and more sexual partners, increased the risk of STI [29]. These findings are supported by our data in which the group of immigrants was larger in the 2006–07 survey than in the 1995–96 survey. Our pooled risk factor analysis in individuals older than 15 years of age confirmed that the increase in HPV seroprevalence over time could partially be explained, in addition to demographic characteristics, by changes in sexual behaviour between the two time periods. After adjustments of demographic characteristics the differences in seroprevalence declines slightly, while a somewhat larger impact was found after adjustment for sexual behaviour. Then HPV seroprevalence between both studies differed only with 0.6%. The largest difference between both serosurveys was found for HPV16 and HPV31, indicating that these HPV types have increased in circulation. We can not exclude that the observed difference was due to variation in selective participation rates between both surveys. However, national data on age of sexual debut are in agreement with our data. The median age of girls who were sexually active decreased from 18 years in 1995 to 16 years in 2006 [30], [31], [32]. The observed shift in the rise in HPV16 seroprevalence to earlier age female cohorts in 2006–07 compared with 1995–96 corresponds with these data. The HPV16 seroprevalence among women aged 15–24 years in the 2006–07 survey was comparable with a previous cross-sectional seroprevalence study among Brazilian primiparous women [33].

The observed HPV seroprevalence in children might be due to vertical or horizontal transmission of HPV, as HPV is also transmittable without having sexual contact [20], [34]. In addition, HPV-specific cross-reactive antibodies against HPV types not detected in our 7-valent VLP-MIA but with a high prevalence in children, e.g. cutaneous HPV types, might account for the observed HPV antibody seropositivity in children.

In older adults (65–74 years of age) sexual activity declines with age although 53% is still sexually active [35]. Interestingly, the higher HPV seroprevalences in elderly in 2006–07 compared with 1995–96 might be due to an increase in sexual activity among older aged individuals because of the development of new therapeutic interventions (i.e. Viagra) that can be used when having sexual problems, especially in men [36]. Older women might be more vulnerable to STI because of post-menopausal changes. Post-menopausal changes can cause atrophy or thinning of mucosa with decreased lubrication that can result in small lesions of the cervix and facilitate the entry of pathogens when these women are sexually active [37]. An increase in STI of chlamydia, gonorrhoea, genital herpes, genital warts and infectious syphilis was noticed in the UK among older aged individuals over time [38] indicating the improved sexual activity in these age cohorts, which is in line with our findings. However, infections appearing later in life might represent the reactivation of latent HPV infection acquired many years earlier [39], [40].

The differences in the questionnaire used in both surveys were a limitation of our study. Because the questionnaire used in 1995–96 survey was extended for the 2006–07 survey, not all questions from the 2006–07 survey matched with those from the 1995–96 survey. Questions that were not included in both questionnaires, such as lifetime number of sexual partners, could have been a better predictor of HPV seropositivity as Vaccarella et al. observed that this variable was associated with HPV16 and 18 seropositivity [41]. Although response rates between both surveys were different, adjustments to the Dutch reference population minimized the bias in seroprevalence and associations with risk variables. Furthermore, serum samples collected in the 1995–96 survey were stored for almost 15 years now at −80°C and this storage time could have affected the quality of the sera. Although we observed an increase in HPV-specific seroprevalence over time, HPV-specific antibody concentrations remained constant between 1995–96 and 2006–07 surveys. Moreover, if storage time had influenced the antibody levels one would expect this to find for all measured HPV types and not just for HPV16 and 31 alone of which the seroprevalence had increased between the 2 surveys. Therefore, it is not likely that the storage time of the sera did influence the amount of HPV-specific antibodies measured and the observed trends in HPV seroprevalence over time are probably better attributed to changes in sexual behaviour and/or increased circulation of the specific HPV types.

To conclude, our data showed that HPV antibody seroprevalences in the general population have increased in The Netherlands over an 11-year period. HPV16 seroprevalences among young women shifted to younger ages over time, probably due to changes in sexual behaviour over the years, in particular the age of sexual debut. Interestingly, the increase in HPV seropositivity in older aged individuals might be caused by a higher percentage of the older aged individuals who are now still sexually active and these age groups are thus more at risk for STI. Serum antibody responses to HPV can persist over time, also after clearance of infection [12]. HPV antibody responses could therefore serve as a marker of cumulative HPV exposure that can be used to compare trends over time [22]. HPV seroprevalence studies, focusing on trends over time, can provide insight into the distribution of HPV types and infection dynamics and will be efficient in monitoring the impact of HPV vaccination in the general population.

## Supporting Information

Figure S1 **HPV antibody concentrations.** HPV16 (A) and HPV18 (B) antibody concentrations (LU/ml) of seropositive individuals in the 1995–96 (blue dots) and in 2006–07 (red dots) surveys. The dark grey lines indicate the geometric mean concentration. For HPV18 significant different antibody concentrations were found in the age cohorts 1–4 (p = 0.04), 50–59 (p = 0.04) and 60–69 (p = 0.02).
(TIF)
