# Supplementary figures and images for: Changes in Antibody Seroprevalence of Seven High-Risk HPV Types between Nationwide Surveillance Studies from 1995–96 and 2006–07 in The Netherlands
Source: PLoS One. 2012 Nov 12;7(11):e48807. doi: 10.1371/journal.pone.0048807 (PMC3495962; doi:10.1371/journal.pone.0048807)

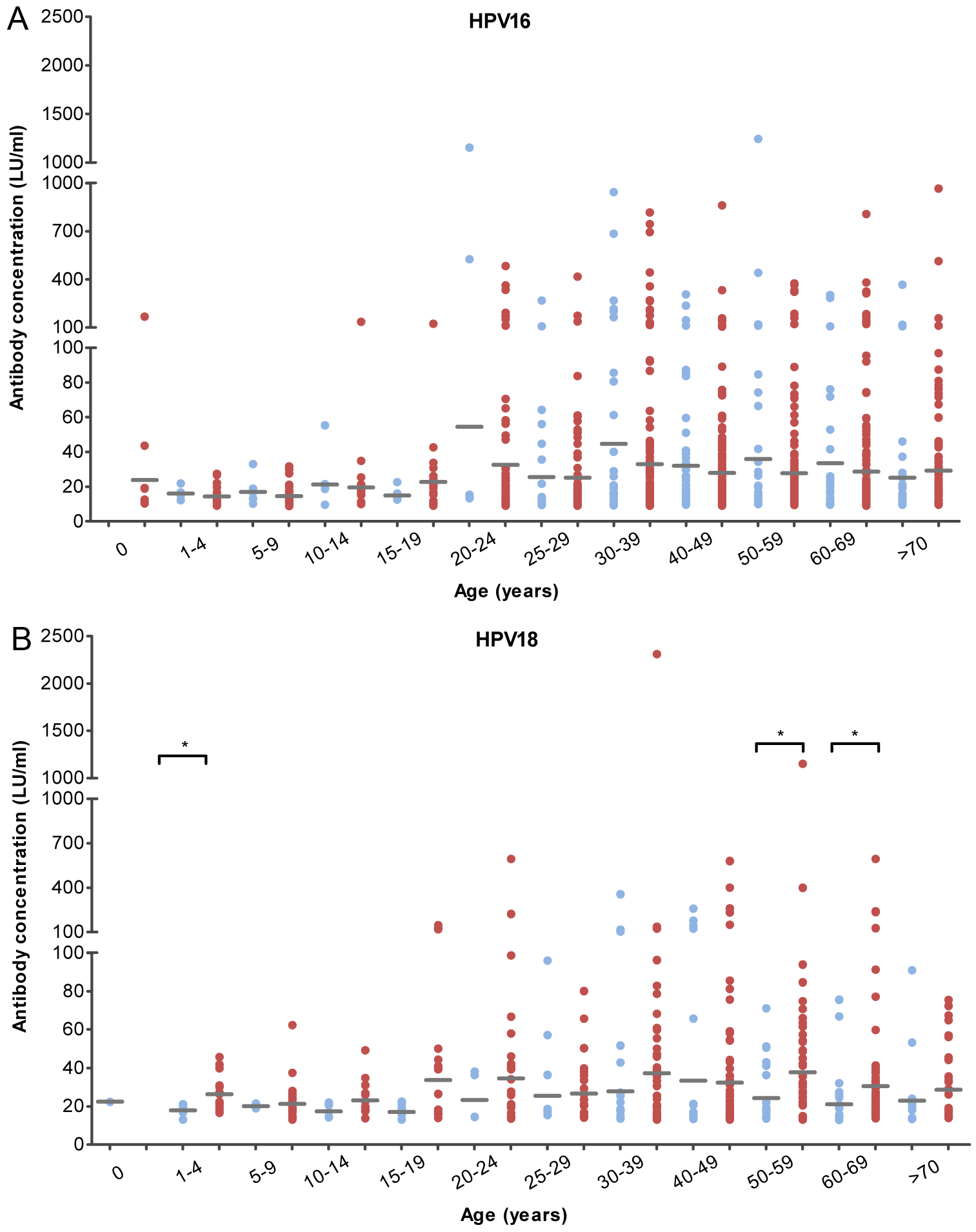

Supplement: Figure S1 — HPV antibody concentrations. HPV16 (A) and HPV18 (B) antibody concentrations (LU/ml) of seropositive individuals in the 1995–96 (blue dots) and in 2006–07 (red dots) surveys. The dark grey lines indicate the geometric mean concentration. For HPV18 significant different antibody concentrations were found in the age cohorts 1–4 (p = 0.04), 50–59 (p = 0.04) and 60–69 (p = 0.02). (TIF) [file pone.0048807.s001.tif]
